# P020. No evidence of microstructural changes in visual network in patients with migraine with aura: a diffusion tensor tract-based spatial statistic (TBSS) study

**DOI:** 10.1186/1129-2377-16-S1-A163

**Published:** 2015-09-28

**Authors:** Antonio Russo, Francesca Conte, Laura Marcuccio, Daniele Corbo, Giuseppina Caiazzo, Alfonso Giordano, Renata Conforti, Fabrizio Esposito, Alessandro Tessitore, Gioacchino Tedeschi

**Affiliations:** Department of Medical, Surgical, Neurological, Metabolic and Aging Sciences, Second University of Naples, Naples, Italy; MRI Research Center SUN-FISM, Second University of Naples, Naples, Italy; Institute for Diagnosis and Care “Hermitage Capodimonte”, Naples, Italy; Neuroradiology Unit, Department of Clinical and Experimental Medicine and Surgery, Second University of Naples, Naples, Italy; Department of Medicine and Surgery, University of Salerno, Baronissi, SA Italy

## Background

Migraine arises from a primary brain dysfunction that leads to episodic activation and sensitization of the trigeminovascular pain pathway. About one third of patients with migraine experiences transient neurological symptoms during attacks, so-called “aura”, among which the most common is visual aura [1]. In our previous fMRI study, we had observed a significantly increased resting-state visual network (RS-VN) functional connectivity in patients with migraine with aura (MwA) compared to patients with migraine without aura (MwoA) during the interictal period [2]. Nevertheless, both whole-brain and visual pathways microstructural white matter (WM) abnormalities in patients with MwA and MwoA is still under debate.

## Objective

To investigate both whole-brain and visual pathways WM microstructural changes in MwA patients, compared to MwoA patients and HC during the interictal period.

## Methods

By using magnetic resonance imaging and diffusion tensor imaging (DTI) with tract-based spatial statistic (TBSS) analysis, we analyzed WM integrity in twenty patients with MwA, compared to twenty patients with MwoA and twenty HC. We performed a TBSS analysis generating fractional anisotropy (FA), mean diffusivity (MD) and radial diffusivity (RD) and axial diffusivity (AD) maps. TBSS was run with FA maps to create the “skeleton”, which represents the center of all fiber bundles in common to all subjects [3]. The resulting statistical maps were thresholded at p < 0.05 corrected for multiple comparisons at a cluster level. Besides whole brain analyses, a region of interest (ROI) analysis was also performed to correlate the TBSS results with both visual pathways standard anatomic ROI data and functional regions that were based on the results of our previous fMRI study.

## Results

Between-groups analyses did not reveal statistically significant differences in both whole-brain and bilateral visual pathways ROI FA, MD, RD and AD values between patients with MwA compared with patients with MwoA and HCs (p < 0.05 corrected).

## Conclusions

Our preliminary data may support the hypothesis that visual pathways functional changes may not be linked to, or alternatively, may precede structural abnormalities in patients with MwA. Furthermore, MwA does not seem to be a risk factor for progressive microstructural WM changes in diffusion tensor tract-based spatial statistic (TBSS) analysis.

Written informed consent to publish was obtained from the patient(s).
